# Youth StepCare: a pilot study of an online screening and recommendations service for depression and anxiety among youth patients in general practice

**DOI:** 10.1186/s12875-019-1071-z

**Published:** 2020-01-07

**Authors:** Belinda Louise Parker, Melinda Rose Achilles, Mirjana Subotic-Kerry, Bridianne O’Dea

**Affiliations:** 1 Black Dog Institute, Prince of Wales Hospital, Hospital Road, Randwick, Sydney, NSW 2031 Australia; 2 Faculty of Medicine, University of New South Wales, High Street, Kensington, Sydney, NSW 2052 Australia

**Keywords:** Stepped care, Youth, Mental health, Screening, Web-based, Primary care

## Abstract

**Background:**

General Practitioners (GPs) are ideally placed to identify and manage emerging mental illness in young people, however, many report low levels of confidence in doing so. A web-based universal screening service delivered via a mobile tablet, Youth StepCare, was developed to assist GPs in identifying depression and anxiety symptoms in youth patients. This service also provided evidence-based treatment recommendations and fortnightly monitoring of symptoms. The current study assessed the feasibility and acceptability of delivering the Youth StepCare service in Australian general practices.

**Methods:**

A 12-week uncontrolled trial was undertaken between August 2018 and January 2019 in two general practices in NSW, Australia. The service was offered to all youth patients aged 14 to 17 years who visited a participating GP during the screening period with their parent or guardian. Youth patients reported the presence of depressive and anxiety symptoms using the self-report Patient Health Questionnaire-9 and the Generalised Anxiety Disorder Questionnaire-7. New cases were defined as those who reported symptoms but were not currently seeking help from their GP, nor had sought help in the past. Feasibility and acceptability among GPs and practice staff were assessed using a battery of questionnaires.

**Results:**

Five GPs and 6 practice staff took part. A total of 46 youth patients were approached, 28 consented, and 19 completed the screener (67.9%). Nine reported symptoms of anxiety or depression, two of which were new cases (22.2%). GPs and practice staff were satisfied with the service, reporting that there was a need for the service and that they would use it again.

**Conclusions:**

The Youth StepCare service appears to be a useful tool for identifying youth with unidentified symptoms of mental illness that can be easily embedded into general practice. Further research would benefit from exploring the factors affecting initial GP uptake and a larger trial is required to determine the efficacy of the service on young people’s symptom reduction.

## Background

General Practitioners (GPs) are a key point of contact with the healthcare system for young people. Given the bi-directional association between mental and physical health [1–5], GPs are well placed to identify emerging mental health problems and intervene early to provide the necessary care [6–8]. This is particularly important for adolescents as their lower mental health literacy and self-awareness means they often rely on others to recognise their changing emotional state and initiate help-seeking [6, 8–10]. However, GPs face several challenges when treating mental health problems among youth. Many GPs report low confidence in identifying, diagnosing, and appropriately managing mental illness among young people due to an absence of specialty training and inadequate time within appointments [11–17]. Combined with the low rates of proactive help-seeking by young people [18–20], these factors reduce GPs capacity to engage with and prioritise mental health for their youth patients [11, 16, 18, 19].

A range of clinical treatment guidelines recommend GPs conduct regular screening and monitoring of patients’ mental health to reduce illness onset and deterioration, and to ensure appropriate and timely treatment [7, 20, 21]. Screening can equip GPs with an effective method to identify symptoms and initiate treatment. This is particularly important for the detection of suicidal ideation, a common symptom of depression, as relying on spontaneous disclosure may lead to underestimations of prevalence and untimely or non-responsive care [22]. Technology offers a useful way to conduct screening in primary care as results can be generated automatically and be made available electronically for review. Decision-making support, such as referral options and psychoeducation, can be easily integrated into these tools to guide GPs’ consultations and treatment decisions. Furthermore, technology allows for repeated screening which improves GPs’ ability to monitor their patients’ symptoms over time.

Technology-based screening services for mental illness have now been implemented in various healthcare settings. For adults, the UK’s *Integrating Mental and Physical healthcare: Research Training and Services (IMPARTS* [23]*)* program and the Australian *StepCare* [24, 25] service provide hospital specialists and GPs with a tool to screen patients’ mental health symptoms prior to their appointment, with results integrated into medical software for immediate review. Both services were found to be acceptable for use among practitioners and patients and feasible to implement within their intended clinical context [23–25]. Two similar screening services have also been trialled in Australia to meet the unique needs of adolescents. Webb et al. [25] examined the acceptability and effectiveness of *Check Up GP* for improving self-disclosure and Reid et al. [26] examined the effectiveness of *mobiletype* for improving mental health outcomes. *Check Up GP* was found to increase the disclosure of sensitive issues, and through up-skilling of GPs, the participants using *mobiletype* experienced substantially improved mental health outcomes overall, demonstrating the usefulness of such screening programs within primary care. However, both services faced major challenges in implementation that have limited their broader uptake and use.

To avoid disruptions to usual ways-of-working, technology delivered screeners need to be embedded into existing workflows in a manner that reflects the ways GPs operate. Both *Check Up GP* and *mobiletype* use external websites that require GPs to access and review, which can be perceived as burdensome. Screening services are also strengthened when decision-making support is provided, particularly for complex health issues like mental illness. Linking GPs to information on clinical treatment guidelines, resources for patients, and treatment recommendations can increase their confidence in managing mental illness [26]. Finally, as adolescents do not visit their GP as frequently as other age groups [27], screening services should be easily accessible, of low intensity, and include non-intrusive monitoring capabilities that do not rely on young people returning to the practice to continually re-screen. Given that the currently available services are not optimised for youth, the Black Dog Institute has developed *Youth StepCare* – a web-based screening service that aims to help GPs identify and treat anxiety and depression in youth patients.

### Aims

The current study aimed to assess the feasibility and acceptability of delivering the *Youth StepCare* service in general practices for youth patients aged 14 to 17 years. Specifically, this study assessed the uptake, need, and operational feasibility of delivering the service, the acceptability and perceived effectiveness among GPs and practice staff, and the barriers and facilitators to its implementation in general practice [28–30]. These outcomes will guide service modifications and improvements to inform future trials of stepped-care services that integrate digital technology and e-health.

## Method

### Design

This study utilised a single arm, uncontrolled pilot design. The study was approved by the UNSW Human Research Ethics Committee (HC180108).

### Study recruitment, consent, and reimbursement

Recruitment of practices and GPs took place between May and August 2018 in New South Wales, Australia. The study advert was sent to practices via Primary Health Networks (Australian government-funded independent-run organisations that coordinate and support primary health services within a specific geographical area) and Black Dog Institute newsletters and mailing lists. Inclusion criteria was the use of Best Practice or Medical Director software, HealthLink Messaging Service, and Wi-Fi internet in the practice. Interested practices were asked to contact the research team, after which a practice visit was arranged. During the visit, the researcher collected signed consent forms, demographics, and information about GP and practice staff interest and training in mental health, and then presented and demonstrated the service in detail and provided training. Following this visit, the researcher completed the General Practice Readiness Questionnaire [24, 31] which was used to determine the level of engagement and readiness of each general practice and staff member for service implementation. At the completion of the study, practices were reimbursed with a gift voucher ($500AUD) and GPs were offered free access to accredited professional development and training (valued at $360AUD). Youth patients were recruited for 93 days, between the 20th August until the 21st November 2018. Practice staff were instructed to offer the tablet to all eligible youth patients who presented to their appointment with their parent. Together with their child, the parent was asked to review the service information and instructions on the mobile tablet and provide their online consent. Youth were eligible to use the service if they were: i) aged between 14 and 17 years; ii) accompanied by a consenting parent or guardian; iii) had a valid mobile phone number or email address; and iv) had the ability to read and speak English. Youth patients who were considered by the GP or practice staff to be too unwell for screening (e.g., vomiting, weak, experiencing psychosis, cognitively impaired) were excluded.

### Service procedure

**Fig. 1:**
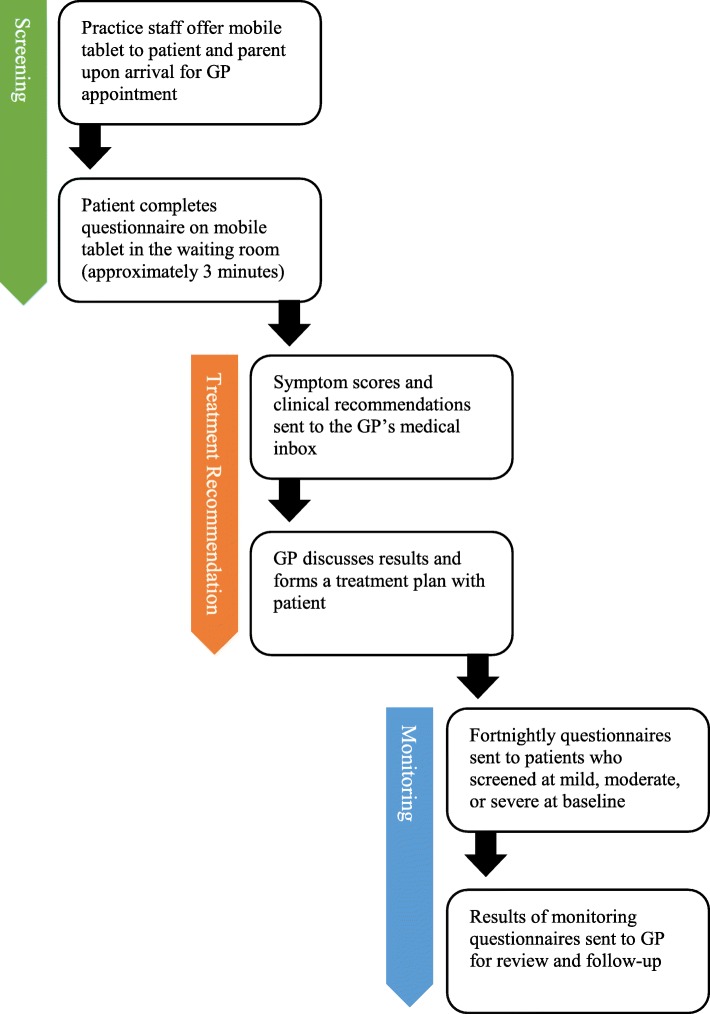
*Youth StepCare* patient and GP journey

The service consisted of three components: i) screening, ii) treatment recommendations, and iii) patient monitoring (Fig. 1).

*Youth StepCare* was delivered to a young person on a mobile tablet while they awaited their GP appointment. Practice staff were instructed to invite all youth patients to use the service regardless of their appointment reason. After providing consent on the mobile tablet, the young person registered using their mobile phone number or email, date of birth, and gender. They were then asked to report whether their current or previous appointments were for mental health reasons. The service then delivered two self-report measures including the Patient Health Questionnaire-9 (PHQ-9 [32]) for depressive symptoms and the Generalised Anxiety Disorder Questionnaire (GAD-7 [33]) for anxiety symptoms. Suicidal ideation was assessed during the initial screener only using participants’ responses to item nine on the PHQ-9 which asked “In the past two weeks, have you been bothered by any thoughts that you would be better off dead or of hurting yourself” rated 0 (not at all) to 3 (nearly every day). Using the highest total score from either scale, the service automatically assigned each patient to one of four treatment steps with treatment recommendations matched to symptom severity (see Table 1). A report with this information was then sent to the GP’s medical software within 3 min via a secure health messaging service. Patients received brief empathic feedback on the mobile tablet which reflected their responses to the screening items (e.g. “it looks like things have been a bit tough for you lately”) and if symptomatic, received help-seeking resources and services (e.g. telephone helplines and websites) and a prompt to talk to their GP in their consult about how they were feeling. All patients were reminded that their results would be immediately shared with their GP. After reviewing the feedback, the patient was then instructed to return the mobile tablet to the practice staff. The fortnightly monitoring surveys were automatically initiated for all symptomatic patients at baseline and delivered via SMS or email (see Table 1). Patients who reported worsening symptoms in the monitoring surveys were advised to schedule an appointment with their GP. GPs also received notifications for any patient who failed to complete the monitoring or who reported that their symptoms had deteriorated, improved, or remained unchanged for four consecutive weeks.

**Table 1:** Youth StepCare treatment model

Step	Symptom Severity	PHQ-9 (GAD-7) score range	Suicidal Ideation	Treatment Recommendation	Monitoring
0	Nil-Minimal	0–4 (0–4)	0	No action required	Not required
1	Mild	5–9 (5–9)	1	Referral to a web-based psychoeducation program	Fortnightly for 12 weeks
2	Moderate	10–19 (10–14)	2	Referral to a psychologist; Consider referral to Child and Adolescent psychiatrist; Referral to web-based psychoeducation program and online cognitive-behaviour therapy (CBT).	Fortnightly for 12 weeks
3	Severe	20+ (15+)	3	Referral to a psychologist or Child and Adolescent psychiatrist; Referral to web-based psychoeducation program and online CBT.	Fortnightly for 12 weeks

### Measures

The schedule of measures is outlined in Table 5 (see Appendix). All measures have been used in prior studies [24, 31] and were adapted for use among GPs and practice staff working with young people.

#### Service uptake

Measured by the proportion of GPs and practice staff who agreed to use the service and the proportion of youth who accepted the mobile tablet from practice staff.

#### Service need

Measured by the number of new cases (i.e. symptomatic youth who had not sought care previously and were not seeing a GP at the current visit for mental health) and the number of GPs who agreed that there was a need for the service.

#### Perceived effectiveness

Measured by the proportion of GPs who followed the treatment recommendations, the proportion of patients who had their treatment modified due to the service recommendations, and improvements in GPs’ ratings of their confidence to provide quality care and ability to identify and monitor their young patients’ mental health (answered on a self-rated 5-point Likert scale ranging from 1 = not at all to 5 = completely).

#### Operational feasibility

Defined as the likelihood of the service being easily embedded into existing workflows and measured by the number of technical difficulties experienced, ratings for how much the service changed usual practice, and how well the service aligned with existing practice software and processes (answered on a self-rated 5-point Likert scale ranging from 1 = not at all to 5 = completely).

#### Acceptability

Defined by satisfaction, likely future use, and practice staff comfort using the service. Satisfaction was measured by rating how satisfied they were with the service and whether it fits with their beliefs and philosophies about general practice (answered on a self-rated 5-point Likert scale ranging from 1 = not at all to 5 = completely). Likely future use was measured by the number who reported they would use the service again in the future and recommend it to others. Comfort was measured by the number of practice staff who stated they were comfortable offering the service to eligible youth.

### Data collection and analysis

The data collected by the service was stored securely on the Black Dog Institute e-health platform hosted on the University of New South Wales servers in Australia. Data was then downloaded into Microsoft Excel and exported to SPSS Version 24.0 (SPSS Inc., Chicago, Il, USA) for analysis. Basic descriptive statistics were conducted and reported for all relevant data.

## Results

### Service uptake and need

Two practices expressed interest in using the service and agreed to take part in the pilot (one rural, one metropolitan). The overall consent rate among GPs was 31.3% (*n* = 5/16 of the GPs at the participating practices agreed to use the service and take part in the study) and 66.7% among practice staff (*n* = 6/9 practice staff at the participating practices agreed to use the service and take part in the study). Table 2 presents the background characteristics of these participants.

**Table 2:** GP and practice staff characteristics

	GPs (*N* = 5)		Practice staff (*N* = 6)	
	*n*	%	*n*	%
Employed full time	3	60.0	6	100.0
Completed training in mental health	5	100.0	0	0.0
Have an interest in mental health	4	80.0	2	33.3

**Fig. 2:**
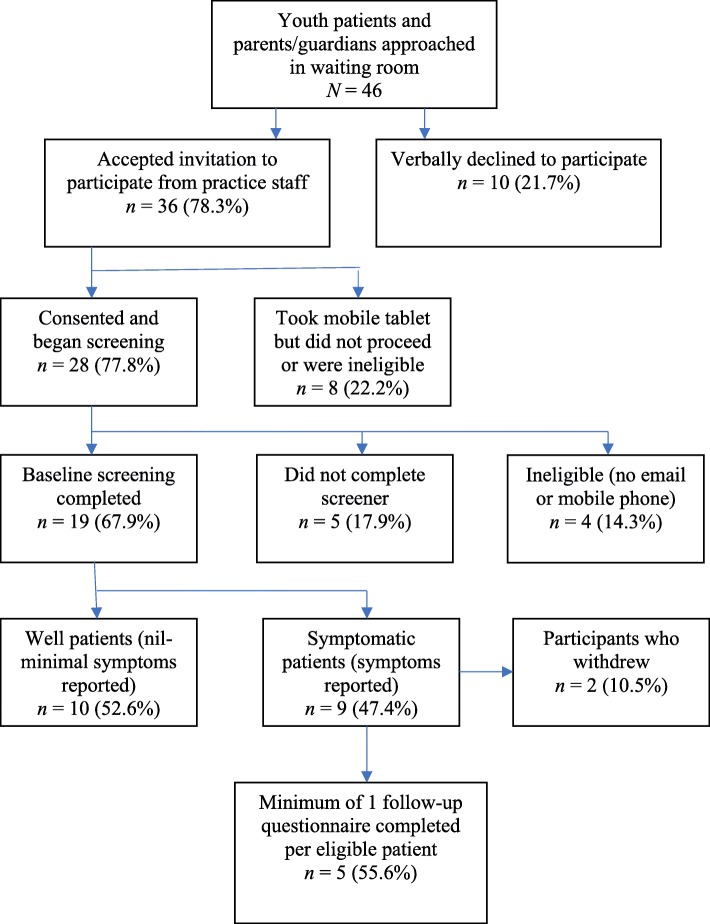
Recruitment and trial process for youth participants

The recruitment flow for youth patients is outlined in Fig. 2. A total of 46 youth patients were offered the service by practice staff, with 36 accepting the mobile tablet (78.3%). Of these, 28 consented and 19 completed the screener (52.8%). Table 3 presents the demographics and mental health history of the final youth sample (mean age = 15.21 years, *SD* = 0.79, range:14–16). In total, 9 youth patients screened as symptomatic for depression and/or anxiety (47.4%, *n* = 9/19). Two patients reported mild symptoms, five reported moderate to moderately severe, and two reported severe symptoms of depression and/or anxiety. Most of the symptomatic patients had seen a GP in the past for mental health reasons (*n* = 7/9; 77.8%). Two were not attending their current appointment nor had seen a GP or other professional previously for mental health reasons and both reported moderate symptoms. On average, the symptomatic youth (*n* = 9) completed 2 monitoring surveys (*SD* = 1.26, range: 1–4). After taking part in the pilot, all GPs surveyed (*n* = 4) agreed that there was a need for the service.

**Table 3:** Characteristics of youth sample (*N* = 19)

	Whole sampl (*N* = 19)		Symptomatic (*n* = 9)	
	*n*	%	*n*	%
Female	13	68.4	7	77.8
Located in regional area	14	73.7	6	66.7
Had previously seen the attending GP	15	78.9	8	88.9
Had previously seen any GP for mental health reasons	12	63.2	7	77.8
Attending the current appointment for mental health reasons	2	10.5	1	11.1

### Perceived effectiveness, operational feasibility, and acceptability

Five GPs completed the baseline survey and four completed the follow-up survey. All six practice staff completed the follow-up feasibility survey. Table 4 presents GP and practice staff responses to the statements regarding perceived effectiveness, operational feasibility, and acceptability of the service. None of the GPs experienced technical problems.

**Table 4:** Participants perceived effectiveness, operational feasibility, and acceptability among GPs and practice staff

Evaluation Domain	Statement	GPs		Practice Staff
		Baseline (*N* = 5) *M (SD)*	Follow-up (*N* = 4) *M (SD)*	Follow-up only (*N* = 6) *M (SD)*
Perceived effectiveness	To what extent do you think Youth StepCare …			
	… helps you to identify young people in need of mental health assistance	4.60 (0.55)	3.75 (0.50)	–
	… helps you to monitor young people’s mental health and respond appropriately	4.40 (0.55)	3.50 (0.58)	–
	… increases your confidence in caring for young people’s mental health	4.20 (0.84)	3.50 (0.58)	–
	… helps improve the quality of mental healthcare provided to young people	4.60 (0.55)	3.67* (0.58)	4.20 (0.75)
Operational feasibility	… changes your usual practice	–	2.75 (1.26)	2.00 (1.27)
	…aligns with your practice’s existing structure and processes	–	4.50 (0.58)	3.70 (1.21)
Acceptability	…fits with your beliefs and philosophies about general practice?	–	4.50 (0.58)	4.20 (0.98)
	Overall, how satisfied are you with Youth StepCare?	–	4.50 (0.58)	4.30 (0.85)
			GPs Agreed *n* (%)	Practice Staff Agreed *n* (%)
	Would use the Youth StepCare service again in the future		4 (100.0)	5 (83.3)
	Would recommend Youth StepCare to other GPs and staff		4 (100.0)	5 (83.3)
	Comfortable offering the mobile tablet to patients and their parents		–	6 (100.0)

*Note.* *Missing data for one participant (*N* = 3)

GPs reported that they followed the treatment recommendations for 4/9 patients, did not follow the treatment recommendations for 1/9, with the remaining four unclear or unknown. One GP reported they provided additional mental health treatment for one patient based on the service recommendations.

## Discussion

The current study assessed the acceptability and feasibility of delivering *Youth StepCare* in general practices within Australia. The success of the trial was determined by uptake and need, acceptance and perceived effectiveness among GPs and practice staff, and the degree to which the service was easily integrated into current practice workflows. Overall, the service was found to be acceptable among the participating GPs and practice staff. These participants were satisfied with the service and reported that they would recommend it to others and use it again. While these findings may not be generalisable to all GPs, these initial positive results, combined with parents’ and young people’s willingness to participate, suggest that a universal screener and treatment recommendations service may be appropriate and feasible for improving the provision of youth mental healthcare among Australian GPs.

### Service need and uptake

The results of this pilot partly confirmed the need for the *Youth StepCare* service. The screener successfully identified two new cases of mental health symptoms in youth patients that may have gone undetected by the GP. At the conclusion of the study period, the participating GPs reported that they believed there was a need for a service of this kind, suggesting they would benefit from services that improve their ability to identify and treat mental illness in youth. The initial interest (78.3%) and uptake (77.8%) in eligible youth patients was high, especially when compared to other trials. For example, less than half of the youth who were approached to use the *Check Up GP* service accepted the invitation [25]. Our higher rates of participation may be due to the youth patients being approached by the practice staff on arrival rather than by the GP (as in *mobiletype*) or over the phone by practice staff (as in *Check Up GP*). When considering the short screening period of this trial, the number of youth screened is equivalent to similar youth studies [25, 34]. Unfortunately, due to the pace of the front-of-practice procedures and lack of access to patient-level data, it was not possible to determine the total number of adolescents who visited the participating GPs during the trial period and were not approached (i.e. those that attended without a parent). Future studies would benefit from obtaining an accurate number of total eligible youth patients and considering an observational design to better understand front-of-practice processes and procedures. Importantly however, these initial results do not provide any evidence to suggest that youth patients and their families were reluctant to engage in the proposed service and requires further investigation.

### Perceived effectiveness

Although the GPs agreed that *Youth StepCare* helped with their identification, monitoring, confidence, and quality of care for youth mental health, perceived effectiveness reduced from baseline to follow-up. This reduction is at odds with GPs’ overall satisfaction of the service and the view that the service aligned with their practice’s beliefs and philosophies. The low number of symptomatic youth patients may have contributed to this as GPs were not able to use the service as extensively as was anticipated. A longer screening period may increase GP’s perceptions of effectiveness. Furthermore, the poor completion of the monitoring surveys by youth resulted in GPs receiving a substantial number of non-adherence reports, which may have negatively impacted their perceptions of effectiveness. However, the high GP scores at baseline across all measures likely represents their enthusiasm about this new and novel service, indicating that the participating GPs have optimistic perceptions about technology-supported screening tools. Future trials would benefit from longer use periods and implementing strategies to increase completion of the monitoring surveys such as customisable time and date reminders. These improvements would present GPs with greater opportunity to experience the benefits of the service.

### Operational feasibility

To overcome some of the implementation barriers experienced by previous screening services, *Youth StepCare* was designed to be implemented into existing practice workflows and systems, requiring minimal set-up and human effort for both GPs and practice staff. A major strength of this service is that it was embedded directly into existing practice software which enabled seamless access for GPs. Delivered in the waiting room, the service did not use appointment time for screening, reducing time pressures on GPs. In contrast to other existing screening services, *Youth StepCare* delivered patients’ results using a standard practice for Australian GPs. This design aspect was well received by GPs, reflected by their high ratings of service alignment. Using familiar processes reduces service complexity and instils the sense that GPs “own” the service rather than researchers [23].

In contrast, practice staff reported that *Youth StepCare* was less aligned with their usual structures and processes. This suggests that the introduction of the mobile screening tablet added to their usual duties. Practice staff were required to increase their interaction with youth patients while also needing to check patients’ age and parental presence for eligibility upon arrival, then offer the mobile tablet (ensuring it was fully charged) and answer any questions the patients may have had. Practice staff did, however, report that implementing the service did not significantly impact their usual practice and that they felt comfortable approaching youth patients. Different results may be found in other practices as the practice staff in the current study were highly enthusiastic towards this research. The additional requirements for practice staff may have a negative impact on those who are less confident and comfortable, do not have as vested an interest in mental health, or do not see the benefit for their GPs and patients. Given general practice organisation, staff commitment to the project, and workflows are barriers to implementation [35, 36], careful consideration needs to be given to the role of the practice staff in the *Youth StepCare* service [37]. Future trials may benefit from providing practice staff with additional training in mental health and nominating champions to lead the implementation of the service on a day-to-day basis. Providing staff with feedback and information on the benefits of the service, including number of people screened and identified, may help build support and enthusiasm for the service.

### Limitations

The current study is one of the first to examine a new and novel youth mental health screening tool for primary care. However, although Australian GPs have a positive attitude towards research [38], their participation rate in the current study was low. While it wasn’t possible to determine the reasons for non-participation, past studies have found that lack of time and funding are major barriers to partaking in research [38–40] alongside workload demands, low levels of confidence or interest in mental health, preference to rely more heavily on clinical experience when making treatment decisions, and disinterest in research [38, 41]. *Youth StepCare* was only compatible with Best Practice or Medical Director software, which may have limited GPs’ participation. Future studies may benefit from implementing specific recruitment strategies to address these factors, collecting reasons for non-participation, and targeting a more diverse sample of GPs. A further limitation of the current study was the inability to fully assess and describe how the service impacted and changed the work activities of practice staff. Future studies would benefit from assessing this aspect given practice staff indicated that the service did not align with their usual duties.

## Conclusions

This current study indicates that this new service, *Youth StepCare*, which screens young patients’ mental health, provides treatment recommendations, and monitors symptoms within general practice may be a promising solution for identifying new cases and improving the quality of GP care provided to Australian youth. This service provides the opportunity for GPs to detect mental health problems early in their progression in a setting where help and care can be provided swiftly and appropriately. Preliminary results indicate that the service was well received and easily embedded into the general practice software and highlights the potential for this type of service to help GPs identify new cases of mental ill-health and prevent further deterioration of existing symptoms. However, low uptake from GPs limits the broader generalisable conclusions that can be drawn. Future trials should consider longer screening periods to increase the number of youths accessing the service, engaging more closely with practice staff to streamline and improve the front-of-practice procedures, and include a larger number of GPs. The next steps for trialling the service should include a direct measure of GP referral behaviour and symptomatic youth access to mental health services and education to better understand how helpful and utilised the service is within the primary care context.

## Data Availability

The datasets used and/or analysed during the current study are available from the corresponding author on reasonable request.

## Appendix

**Table 5:** Schedule of measures

Measure	Baseline	2, 4, 6, 8, 10, week follow-up	12-week endpoint
General Practice			
Readiness Questionnaire	X		
General Practitioners			
Demographic and Background Questionnaire	X		
Feasibility Baseline Questionnaire	X		
Clinical Recommendations Form	X		
Feasibility Follow-up Questionnaire			X
Practice Staff			
Feasibility Questionnaire			X
Youth Patients			
Demographic and Background Questionnaire	X		
Patient Health Questionnaire (PHQ-9; PHQ-8 from week 2 onwards)	X	X Only those who score ≥ 5 at baseline	X
Generalised Anxiety Disorder Scale (GAD-7)	X	X Only those who score ≥ 5 at baseline	X
Functioning Question	X	X Only those who score ≥ 5 on PHQ-9 or GAD-7 at baseline	X
Patient Adherence Questionnaire		X Only those who score ≥ 5 on PHQ-9 or GAD-7 at baseline	X

## Abbreviations

AUD: Australian Dollars
CBT: Cognitive behavioural therapy
GAD-7: Generalised Anxiety Disorder Questionnaire 7-item
GPs: General Practitioners
IMPARTS: Integrating Mental and Physical healthcare: Research Training and Services
NSW: New South Wales
PHQ-9: Patient Health Questionnaire-9
SPSS: Statistical Package for Social Sciences
